# Assessment of artemisinin tolerance in *Plasmodium falciparum* clinical isolates in children with uncomplicated malaria in Ghana

**DOI:** 10.1186/s12936-023-04482-w

**Published:** 2023-02-19

**Authors:** Samuel Yao Ahorhorlu, Neils Ben Quashie, Rasmus Weisel Jensen, William Kudzi, Edmund Tetteh Nartey, Nancy Odurowah Duah-Quashie, Felix Zoiku, Bartholomew Dzudzor, Christian William Wang, Helle Hansson, Michael Alifrangis, George Obeng Adjei

**Affiliations:** 1 Centre for Tropical Clinical Pharmacology and Therapeutics, University of Ghana Medical School, University of Ghana, P.O. Box 4236, Accra, Ghana; 2 Centre for Medical Parasitology, Department of Immunology and Microbiology, University of Copenhagen, Copenhagen, Denmark; 3 Department of Infectious Diseases, Copenhagen University Hospital (Rigshospitalet), Copenhagen, Denmark; 4 Department of Epidemiology, Noguchi Memorial Institute for Medical Research, University of Ghana, Accra, Ghana; 5 Department of Medical Biochemistry, University of Ghana Medical School, University of Ghana, Accra, Ghana; 6 West African Centre for Cell Biology of Infectious Pathogens, University of Ghana, Accra, Ghana

**Keywords:** Artemisinin tolerance, *Plasmodium falciparum*, Uncomplicated Malaria, Ex vivo ring-stage survival assay, *Kelch 13*, *Pfcoronin*, Ghana

## Abstract

**Background:**

Artemisinin-based combination therapy (ACT) is the first-line treatment for uncomplicated malaria in Ghana. Artemisinin (ART) tolerance in *Plasmodium falciparum* has arisen in Southeast Asia and recently, in parts of East Africa. This is ascribed to the survival of ring-stage parasites post treatment. The present study sought to assess and characterize correlates of potential ART tolerance based on post-treatment parasite clearance, ex vivo and in vitro drug sensitivity, and molecular markers of drug resistance in *P. falciparum* isolates from children with uncomplicated malaria in Ghana.

**Methods:**

Six months to fourteen years old children presenting with acute uncomplicated malaria (n = 115) were enrolled in two hospitals and a Health Centre in Ghana’s Greater Accra region and treated with artemether-lumefantrine (AL) according to body weight. Pre- and post-treatment parasitaemia (day 0 and day 3) was confirmed by microscopy. The ex vivo ring-stage survival assay (RSA) was used to detect percent ring survival while the 72 h SYBR Green I assay was used to measure the 50% inhibition concentration (IC_50s_) of ART and its derivatives and partner drugs. Genetic markers of drug tolerance /resistance were evaluated using selective whole genome sequencing.

**Results:**

Of the total of 115 participants, 85 were successfully followed up on day 3 post-treatment and 2/85 (2.4%) had parasitaemia. The IC_50_ values of ART, artesunate (AS), artemether (AM), dihydroartemisinin (DHA), amodiaquine (AQ), and lumefantrine (LUM) were not indicative of drug tolerance. However, 7/90 (7.8%) pre-treatment isolates had > 10% ring survival rates against DHA. Of the four isolates (2 RSA positive and 2 RSA negative) with high genomic coverage, *P. falciparum (Pf) kelch 13* K188* and *Pfcoronin* V424I mutations were only present in the two RSA positive isolates with > 10% ring survival rates.

**Conclusions:**

The observed low proportion of participants with day-3 post-treatment parasitaemia is consistent with rapid ART clearance. However, the increased rates of survival observed in the ex vivo RSA against DHA, maybe a pointer of an early start of ART tolerance. Furthermore, the role of two novel mutations in *PfK13* and *Pfcoronin* genes, harboured by the two RSA positive isolates that had high ring survival in the present study, remains to be elucidated.

**Supplementary Information:**

The online version contains supplementary material available at 10.1186/s12936-023-04482-w.

## Background

Artemisinin (ART) resistance or more appropriately tolerance, in *Plasmodium falciparum* has been reported mostly in Southeast Asia (SEA) and partly in East Africa [1–3]. This is a cause for concern as any widespread resistance to ARTs and artemisinin-based combination therapy (ACT) in the absence of other potent, safe, and effective anti-malarial drugs, could reverse the clock of progress in the fight against global malaria. This will consequently be dire for sub-Saharan Africa (SSA) where the disease is most prevalent and a major public health burden [4].

Taking a cue from the devastating effects of *P. falciparum* resistance to chloroquine, which originated simultaneously from SEA and South America [5] and spread to East Africa and then to other parts of SSA, monitoring and surveillance of parasite dynamics in drug susceptibilities are important. These strategies remain key in detecting subtle changes that could eventually lead to ART tolerance/resistance in local malaria parasite populations, although currently clinically indiscernible. Dondorp et al. [2] described the observation of a subset of surviving ring-stage *P. falciparum* parasites following treatment with artesunate (AS) monotherapy or ACT as delayed parasite clearance. This observation was subsequently made by other authors [6, 7]. However “true” artemisinin resistance remains to be confirmed [8]. Recent in vitro and in vivo studies have postulated molecular grounds for delayed parasite clearance by implicating several mutations in the *P. falciparum* kelch 13 (*Pfk13*) propeller domain [2, 9–11]. The World Health Organization (WHO) Global Malaria Programme Status Report on ARTs and ACT efficacy 2018, defines partial ART resistance as delayed parasite clearance following treatment with an artesunate monotherapy or with an artemisinin-based combination therapy (ACT) [8]. It states that *Pfk13* mutations are validated as markers for artemisinin resistance when such mutations are correlated with slow clearance in clinical studies, are correlated with reduced in vitro drug sensitivity, such as ring stage survival assays (RSA) using fresh isolates, or when an insertion of the K13 mutant in transfection studies results in reduced in vitro drug sensitivity [8].

Studies conducted in East African countries, i.e., Rwanda, and Uganda, have revealed a recent independent emergence of partial ART resistance with the observation of increased number of isolates with confirmed *Pfk13* mutations, R561H and A675G, respectively in the two countries [12, 13]. Though ACT continue to be effective in these countries with no immediate consequence for patients, there is apprehension that this partial ART resistance could mediate ACT partner drug resistance spread in the WHO African Region [4].

The ART-tolerant *P. falciparum* phenotype, however, appears to be evolving and so the above characterization may not be conclusive in describing the emerging tolerance. For instance, there are reports of *Pfk13*-independent ART or ACT tolerance [14–16]. Witkowski et al. [17] and Ye et al. [18] established that ex vivo ring stage survival assay (RSA) threshold of 10% ring survival was closely associated with delayed parasite clearance after drug treatment and considered this a surrogate for the ART-resistant phenotype. Presently in Ghana, there is no clinically reported ART resistance. However, the prospect of low-key changes taking place in the local parasite population which could eventually lead to the emergence of ART-tolerance cannot be ruled out. Local evolutionary conditions of ART tolerance may vary between geographical regions [19]. Continuous search for potential predictors of early onset of parasite tolerance to ART and its derivatives in endemic settings such as Ghana is therefore warranted. Thus, this study sought to assess and characterize correlates of potential ART tolerance based on posttreatment parasite clearance, ex vivo and in vitro drug sensitivity, and genetic markers of tolerance/resistance in parasite isolates from children with uncomplicated malaria in Ghana’s Greater Accra region.

## Methods

### Study design

This was a longitudinal, single-arm, prospective study to evaluate *P. falciparum* tolerance to ART and its derivatives in children with uncomplicated malaria aged 6 months to 14 years in 3 health facilities in the Greater Accra region of Ghana. The study focused mainly on day 3 post artemether-lumefantrine (AL) treatment parasitaemia, 72-h ex vivo RSA after dihydroartemisinin (DHA) exposure, 72-h parasite clearance in vitro against a panel of 6 drugs (ART, AS, artemether [AM], DHA, amodiaquine [AQ], lumefantrine [LUM], and the following molecular markers of drug tolerance / resistance: Single Nucleotide Polymorphisms (SNPs), Multiple Nucleotide Polymorphisms (MNPs), Insertions & Deletions (INDEL) in *Pfk13*, *Pfcoronin*, *P. falciparum* multidrug resistance protein 1 (*Pfmdr1)*, multidrug resistance protein 2 (*Pfmdr2)*, dihydrofolate reductase (*Pfdhfr)*, dihydropteroate synthetase (*Pfdhps)*, signal peptide peptidase* (Pfspp)*, and multidrug resistance-associated protein 2 (*Pfmrp2)* genes. It sought to set up correlates of ART tolerance.

### Study sites

This research was carried out in three health facilities in the Greater Accra Region of Ghana: (i). Princess Marie Louise Children’s Hospital [PMLCH], Accra; (ii). Danfa Health Centre [DHC], La Nkwantanang-Madina District; and (iii). Shai Osudoku District Hospital [SODH], Dodowa). The PMLCH is a 74-bed capacity government hospital that offers primary and specialist paediatric care [20]. The SODH, is a 125-bed capacity district hospital located at Dodowa. The DHC is a health center that provides primary healthcare service to a population of 11 000 people in the La Nkwantang district. These health facilities are situated in the Southeastern region of the country characterized by nine months of malaria transmission with increasing incidence from May to November. The laboratory work (in vitro and molecular assays) were done in the Noguchi Memorial Institute for Medical Research, University of Ghana and the Centre for Medical Parasitology, University of Copenhagen, Denmark. Whole genome sequencing was done at the Department of Health Technology, at the Danish Technical University (DTU), Lyngby, Denmark.

### Study population

The study involved 115 Ghanaian children with uncomplicated malaria per WHO criteria [21] aged 6 months to 14 years presenting with fever (axillary temperature > 37.5 °C) measured at the time of admission, parasitaemia in the range of 1000 to 250,000 per microliter of blood, with no known incidence of hypersensitivity reactions to AL, after obtaining written informed consent from the accompanying parent or guardian in children below 12 years of age. Assent was also obtained in addition to parental consent for those older than 12 years. Children with severe malaria, malnutrition, anaemia (haemoglobin < 8 g/dL), or positive RDT but negative microscopic examination for malaria were excluded from the study. Participant enrolment was carried out between June and November 2018. Socio-demographic and relevant clinical data were collected from each participant and 5 mL of venous blood was obtained (prior to AL treatment) for the laboratory investigations and other assays.

### Detection of day 3 post-treatment parasitemia

Post-treatment day 3 parasitaemia was evaluated via light microscopy under oil-immersion. Briefly, both thin and thick blood smears were made and stained with 10% Giemsa-stain after air-drying the methanol-fixed thin smear. Detection of parasite load per microlitre of blood was achieved by the count of parasites per 200 white blood cells (WBCs), or 500 WBCs (if there were less than 10 parasites per field), times 40 or 16 apiece per standard protocol i.e., assuming 8, 000 WBC per µL of blood [22]. The slides were read by two microscopists independent of each other and results were recorded as positive when both microscopists recorded a specimen as positive. Provision was made for a third microscopist should the readings of the two be discordant, for quality control purposes.

### Detection of percent ring-stage survival rates

Percent ring-stage parasite survival was detected by carrying out ex vivo RSA on newly obtained *P. falciparum* field parasites (see supplementary information for details) as previously presented by Witkowski et al. [23]. In brief, parasite density was lowered to 1% and the haematocrit adjusted to 2% for every sample, with uninfected O + red blood cells (RBCs). Parasite count was determined and noted as ‘initial parasitemia’. The test was performed in a 48-well plate with a well with 700 nM DHA and another well without DHA (contains 0.1% dimethyl sulfoxide, [DMSO]) as control well (see full details in supplementary information). To both wells was added *P. falciparum*-infected RBC suspension in RPMI 1640 medium supplemented with 0.5% Albumax, as replacement for inactivated human serum. The contents were gently mixed, after which the plates were gassed (2.03% O_2_, 5.53% CO_2_, and 92.44% N_2_) and incubated at 37 °C for exactly 6 h. The samples were then washed in RPMI 1640 medium for excess drug removal. Complete medium (see supplementary information for exact composition) was used to again mix the cells and the mixture was incubated for 66 h under the same conditions. To differentiate viable and dead parasites, microscopic examination under 100 × oil-immersion was carried out on a thin blood smear made 72 h after drug exposure. Viable parasites were quantified according to the method described by Witkowski et al. [23]. Growth rate was computed as parasites count from the non-exposed well/initial parasitaemia, while percent ring survival (%) was computed as parasite count from DHA-exposed well/parasitaemia from DHA non-exposed well) × 100. Percent survival rates were only explicable for growth rate of 1 or more. Detailed information on how the parasite suspension and assay reagents were prepared is provided (see supplementary information).

### Detection of molecular markers of ART tolerance

DNA was extracted from malaria positive pre-treatment whole blood samples using QIAamp 96 DNA Blood kit (QIAGEN, Hilden, Germany) per the manufacturer’s protocol. Selective whole genome sequencing (sWGS) was done (Department of Health Technology, Danish Technical University (DTU), Lyngby, Denmark) for a set of isolates with > 10% ring survival (n = 4) and selected isolates which were sensitive to DHA (n = 3) in the ex vivo RSA to detect molecular markers of ART tolerance in *P. falciparum.* Single nucleotide polymorphisms (SNPs) in the following molecular markers were analysed: *P. falciparum* kelch 13 (*Pfk13*), *Pfcoronin*, multidrug resistance 1 (*Pfmdr1*), *P. falciparum* multidrug resistance 2 (*Pfmdr2*), *P. falciparum* dihydropteroate synthetase (*Pfdhps*), *P. falciparum* dihydrofolate reductase (*Pfdhfr*), gene expressing signal peptide peptidase and multidrug resistance-associated protein 2 genes. The 3D7 reference genome (GCA_000002765.3, Sep 01, 2020, Mar 01, 2022) was used for the comparative analysis to detect the SNPs. Briefly, selective PCR was carried out using 10 short oligonucleotide primers of 8–12 mers as primers and Phi29 (Φ29) DNA polymerase (extracted from *Bacillus subtilis* phage) to increase the genome of selected parasites (without the telomeric regions) for the sWGS following the protocol detailed by Oyola et al. [24]. Initially, the FastQC was used to quality-assess the Illumina paired-end sequencing reads for the gene variation analysis. Trimming of the reads was done via a Phred ≥ 20 quality score after which comparison of the reads was made to the 3D7 laboratory clone reference genome using the Interactive genome viewer version 2.6.3 (IGV). For each selected gene, the matching location of the genome and gene ID were verified in the PlasmoDB database (PlasmoDB rel. 9.0, 2012-MAY-18). From the IGV each gene was inspected for SNPs, deletions, or insertions in comparison to the 3D7 reference genome. Only genes that had an adequate coverage were analysed, and each verified SNP was converted into amino acid sequence and recorded for each ART “tolerant” or sensitive isolate.

Supplementary information on primer sequence, the PCR reaction mix, the PCR thermal cycling conditions, and an image of the gel of the PCR products of the isolates of interest for the sWGS (this figure is part of the results, not materials and methods) is provided in Table ST1, 2, & 3 and Supplementary Figure SF1.

### Detection of in vitro tolerance of ART and its derivatives and partner drugs

The 50% inhibitory concentrations of ART, AS, AM, DHA, LUM, and AQ (IC_50_) were determined for isolates of interest by the SYBR Green I fluorescence-based in vitro drug susceptibility assay previously described by Johnson et al. [25]. Briefly, parasite mixture (fresh isolates) was added to pre-dosed plates (see supplementary information for details) with the test drugs, placed in a Hypoxia Incubation Chamber (StemCell Technologies Inc., San Diego; USA), gassed (2.03% O_2_, 5.53% CO_2_, and 92.44% N_2_) and incubated at 37 °C for 72 h as previously reported by Quashie et al. [26]. The 3D7 laboratory reference clone served as internal control. Hundred microlitres (100 μl) SYBR Green contained in Malaria SYBR Green 1 fluorescent (MSF) lysis buffer (see supplementary information for full details) was added to each well and the content thoroughly mixed. The 96-well culture plate was wrapped in aluminium foil and kept in the dark for 2 h, after which the microtiter plate reader was used to measure fluorescence. The Worldwide Antimalarial Resistance Network (WWARN) online automated “in vitro analysis and reporting tool (IVART)” [27] and GraphPad Prism version 7.0 (GraphPad Software, San Diego, CA) were used to calculate the IC_50_ values. The geometric mean IC_50_ for the combined data from the study sites was determined for the six selected drugs. The IC_50_ threshold values from literature denoting resistance employed in the present study were as follows: 20 nM for AS, 30 nM for AM, 12 nM for DHA, 12 nM for ART, 80 nM for AQ, and 150 nM for LUM [25, 28, 29]. All assays were performed in triplicate. Information on the preparation of the drugs and the blood parasite mixture is included in the Supplementary Information.

### Data analysis

Statistical analysis was carried out using GraphPad Prism version 7.0 (GraphPad software). Data for the IC_50_ values were first transformed logarithmically, analysed, and reported as geometric mean of the IC_50_ together with their 95% confidence interval. Percent ring stage parasite survival was evaluated by parasite-infected cells count in 10,000 RBCs as compared with survival of parasites in the non-DHA exposed well.

The Spearman correlation test was employed to detect any association that may exist between the ex vivo RSA sensitive and tolerant isolates and potential resistance mutations from the sWGS. Association between percent ring survival and the IC_50_ values of the test drugs was evaluated by the Pearson correlation analysis. The association is deemed significant at a *P*-value below 0.05.

## Results

The median age, temperature, haemoglobin, and parasitaemia of the 115 study participants were 72 months, 38 °C, 10.3 g/dl, and 39,014 parasites/µl, respectively (Table 1). A total of fifty-five (n = 55, 47.8%) study participants were females. Children < 5 years (i.e., 6–59 months) constituted majority of the study participants (51/115, 44.3%). There were 45/115 (39.1%) children aged 10 to 14 years and 19/115 (16.5%) children aged between 5 and 9 years (Table 1).

**Table 1:** Descriptive clinical and demographic data of research subjects

Parameter	N (115)	Median	IQR%
Age (months)		72	36–96
Weight (Kg)		18	12–25
Temperature (^o^C)		38	37.5–39.1
Parasitaemia /µl		39,014	19,168–105,120
Haemoglobin (g/dl)		10.3	9–11.5
Sex	Male	Female	
	60	55	
Age range (months)			Total
Less than 60	27	24	51
60 to120	10	9	19
121 to 168	23	22	45

*N* denotes total number of participants, *IQR* denotes interquartile range

Out of the 115 study participants, 85 (73.9%) of them were successfully followed up to day 3. Of the 85 participants, 83 (97.6%) had negative parasitaemia by microscopy on day 3 while two (2.4%) participants still harboured parasites.

### Detection of percent ring-stage survival rates

Ex vivo RSA results for freshly collected malaria-positive pretreatment whole blood samples against DHA were successfully obtained for 78.3% (90/115) of the study participants. Twenty (17.4%) samples were unsuccessful for the ex vivo RSA on account of low blood volumes and low parasitaemia (1008 parasites/µl – 3168 µl). Five samples (4.3%) had contamination and were discarded. The control sample was an ART-susceptible 3D7 laboratory strain. At a DHA concentration of 700 nM, there was no ring survival in the 3D7 control, and this was similar for 92.2% (83/90) of the clinical isolates. However, seven of 90 (7.8%) isolates had percent survival rate > 10% (i.e., between 10 and 20%).

### Detection of molecular markers of ART tolerance

Seven *P. falciparum* isolates were selected for whole genome sequencing, four (D10, D30, P11, and P55) of which had > 10% ring-stage survival rates and three isolates (P32, P36, P45) with no ring survival. Genotyping of three of the RSA negative isolates (P52, S22, S19) was not successful. Admissible high genomic coverage was obtained for D10 and D30 RSA positive isolates and P32 and P36 RSA negative isolates. P11 and P55 RSA positive isolates had very low genomic coverage, while P45 RSA negative isolate had virtually no coverage (data not shown). This could be due to the samples not having ≥ 180 *P. falciparum* genomes (parasitaemia threshold ~ 0.03%, or ~ 40 parasites per 200 WBC [24]. Sequences from the four isolates, D10, D30, P32, and P36, with high genomic coverage were then compared to the 3D7 reference genome with focus on known malaria resistance marker genes: *Pfk13*, *Pfcoronin*, *Pfmdr1*, *Pfdhfr*, and *Pfdhps*, (the latter two with no relationship to artemisinin resistance). The distribution of SNPs, insertions, and deletions (INDELs) in selected drug resistance genes for isolates with admissible high genomic coverage is presented in Table 2. V424I missense mutation in the *Pfcoronin* gene was harboured by the RSA-positive isolates D10 and D30. The N86Y, D650N, and N652D mutations in the *Pfmdr1* gene, and N245S in the *Pfmdr2* gene were observed in isolate D10. The D30 isolate had D650N and D1951N mutations in the *Pfmrp2* gene (Table 2).

**Table 2:** Distributions of SNPs/MNPs/INDELs of *P. falciparum* kelch 13 (*PfK13*), *Pfcoronin*, multidrug resistance protein 1(*Pfmdr1*), multidrug resistance protein 2 (*Pfmdr2*), dihydropteroate synthetase (*Pfdhps*), dihydrofolate reductase (*Pfdhfr*), multidrug resistance-associated protein 2 (*Pfmrp2*), and signal peptide peptidase (*PfSPP*) genes for D10 and D30 (RSA Positive) and P32 and P36 (RSA Negative) isolates and their frequencies

Sample ID		D30	D10	P36	P32	SNPs/INDELs total
Gene name	SNPs/INDELs					
*PfCoronin*	S183G	1		1		2
	*V424I*	1	1			2
*PfK13*	K189T		1	1	1	3
	K188*	1				1
*Pfmdr1*	Y184F		1		1	2
	*D650N*		1			1
	*N86Y*	1				1
	*N652D*		1			1
*Pfmdr2*	S208N	1	1		1	3
	F423Y	1	1		1	3
	*N245S*		1			1
	I492V		1		1	2
	Del (ATT/ASN)	1	1	1	1	4
*Pfdhfr*	S108N	1	1	1	1	4
	N51I	1	1	1		3
	*D212I*	1				1
	C59R	1	1	1		3
*Pfdhps*	I431M			1		1
	A613S			1	1	2
	S436A		1	1	1	3
	A581G			1		1
*SPP*	*Insert (T)*		1			1
*Pfmrp2*	L199V	1	1	1	1	4
	D634N	1		1	1	3
	D650N	1				1
	N642D	1		1	1	3
	*D1951N*	1	1			2
	K714I	1		1	1	3
	L295V				1	1
	L1531I	1		1	1	3
	S1527T	1	1	1	1	4
	K1842I				1	1
	S1371N				1	1
	D631G		1	1	1	3

*STOP codon, italicized SNPs refer to those that are novel in the Ghanaian population. *SNP* Single nucleotide polymorphism, *MNP *multiple nucleotide polymorphism, *INDEL* Insertions & Deletions. D10 & D30 are isolates with > 10% RSA values.

Detection of in vitro ARTs and partner drugs tolerance

In vitro drug sensitivity testing to determine the IC_50_ of ART and its derivatives as well as partner drugs was performed in triplicates for D10 and D30 (RSA positive) and P32, P52, S22 and S19 (RSA negative) field isolates. The 3D7 laboratory strain was used as negative control (Table 3). The IC50s of ART sensitive isolates for DHA (4.55 nM, 5.18 nM, 3.57 nM, 6.70 nM) were comparable to those of the 3D7 control (4.08 nM) and that of one of the ART tolerant isolates D30 (5.35 nM). One of the ART-tolerant isolates, D10, however, had an IC_50_ of 11.78 nM. The geometric mean IC_50_ values and their corresponding 95% CI for the drugs of interest were 7.05 nM (4.50–10.9), 6.49 nM (5.30–7.90), 7.11 nM (6.30–7.90), 5.73 nM (3.70–8.80), 11.77 nM (9.10–15.3), and 13.62 nM (10.5–17.7) for ART, AS, AM, DHA, LUM, and AQ, respectively.

**Table 3:** In vitro drug sensitivity of two RSA positive and four RSA negative *P. falciparum* isolates to artemisinin, artesunate, dihydroartemisinin, artemether, amodiaquine and lumefantrine

Test drugs (IC50 nM, 95% CI)							
	ART	AS	DHA	AM	LUM	AQ	RSA%
3D7	4.58 (4.17–5.00)	2.75 (2.66–2.85)	4.08 (3.76–4.41)	4.60 (4.38–4.82)	8.52 (7.49–9.55)	7.16 (6.40–7.93)	0.0
D30	4.65 (3.19–6.11)	6.17 (5.08–7.25)	5.35 (3.76–6.93)	7.23 (5.92–8.55)	15.71 (13.43–17.98)	11.8 (8.91–14.69)	13.0
D10	10.81 (9.69–11.94)	9.28 (8.22–10.35)	11.78 (10.31–13.25)	7.21 (6.33–8.10)	10.28 (9.59–10.96)	11.87 (10.83–12.91)	19.0
P52	12.93 (11.22–14.65)	6.64 (6.19–7.10)	5.18 (4.16–6.20)	5.95 (5–60-9.00)	9.16 (8.79–9.53)	11.4 (10.9–11.89)	0.0
P32	6.43 (5.35–7.5)	6.06 (5.37–6.74)	4.55 (3.42–5.68)	7.49 (6.03–8.95)	16.43 (14.44–18.41)	15.02 (12.41–17.63)	0.0
S22	5.75 (4.38–7.12)	6.33 (5.10–7.56)	6.70 (5.93–7.46)	6.71 (5.41–8.01)	11.26 (10.46–12.06)	21.78 (18.54–225.0)	0.0
S19	5.09 (2.95–7.23)	5.13 (3.5–6.77)	3.57 (2.29–4.85)	8.31 (6.11–10.50)	9.73 (7.9–11.57)	12.23 (8.9–15.55)	0.0

*ART* artemisinin, *AS* artesunate, *DHA* dihydroartemisinin, *AM* artemether, *LUM* lumefantrine, *AQ* amodiaquine. D10 & D30: RSA positive isolates; P52, P32, S22, S19: RSA negative isolates, 3D7: laboratory control strain. NB: All assays were done in triplicate

There was no significant relationship between percent ex vivo ring-stage-survival rates and IC_50_ of DHA for the six selected isolates (r_s_ (4) = 0.67, *p* = 0.200).

## Discussion

Ghana remains one of eleven countries with the highest burden of malaria in SSA [30]. The emergence of ARTs tolerance in SEA and parts of East Africa poses a new threat to the fight against malaria in Ghana and elsewhere. Early detection and evaluation of tolerant strains in local parasite populations could help in containment efforts to avert treatment failures. This study was conducted to assess and characterize correlates of potential ART tolerance based on post-treatment parasite clearance, ex vivo and in vitro drug sensitivity, and molecular markers of parasite isolates from children with uncomplicated malaria in the Greater Accra Region of Ghana. Day 3 parasitaemia was detected following AL treatment in only two of 85 participants, and this is consistent with rapid ART clearance in the studied parasite population. This largely agrees with findings from under 5 years children with uncomplicated malaria in studies from Ghana and Nigeria which all reported day 3 positive parasitaemia below 5% [31, 32]. WWARN earlier proposed a day 3 positive parasitaemia of 5% threshold as more suitable in keeping track of ART resistance in SSA instead of the currently recommended 10% by the WHO due to high acquired immunity against the parasite in African populations and its contribution to faster parasite clearance [33].

The present study found high ring stage survival rates > 10% in 7/90 (7.8%) while most of the isolates (83/90, 92.2%) had no surviving rings after exposure to 700 nM DHA. ART-sensitive 3D7 reference strain was used as control and had no ring survival post exposure to 700 nM DHA. The findings of the present study are similar to the results (2.1% isolates with > 10% survival rates) obtained in a Ugandan *P. falciparum* population in 2018 by Ikeda et al. [34]. Earlier, Witkowski et al. identified slow-clearing infections in eastern Cambodia by strongly correlating > 10% ex vivo ring stage survival rates with in vivo parasite clearance half-lives > 5 h (where an isolate with 12.2% ring stage survival had a half-life of 8.17 h). They proposed this to be a proxy for ART-resistance [23]. Therefore, the seven clinical isolates with > 10% ex vivo ring survival rates in this study, could indicate a signal for potential onset of ART tolerant *P. falciparum* strains in a portion of the studied population.

Prior reports proposed strong correlations between mutations in the *Pfk13* propeller domain and ring survival rates [11, 35]. For instance, one of the isolates with > 10% survival rates in a Ugandan population was reported by Ikeda et al. as harbouring A675V, a candidate marker of ART resistance [34]. Mutations in the *Pfk13* > 440 to 680 coding region (propeller domain) which have been associated with ART resistance in SEA, were not detected in the present study. Nonetheless, D10, (an isolate with high ring survival) harboured a PfK188* nonsense mutation which is a novel mutation. Previous reports by Cowell et al. suggest that some nonsense mutations in the *P. falciparum* isolates resulting in stop-gained mutations may play drug detoxification roles [36]. Ariey and colleagues also observed an in vitro selection of a cysteine proteinase-2a gene (S69*) mutation in an African isolate after ART selection pressure [37]. However, the role of PfK188* coupled with background mutations in ring survival remains to be verified.

Though the present study could not establish implicated variations in the *Pfk13* propeller domain for isolates with high ring survival in this present work, the potential evolution of ART-tolerant *P. falciparum* cannot be ruled out in the Ghanaian *P. falciparum* parasite population. This assertion is validated by accounts of isolates with as high as 19.4% ring survival rates without K13 mutations in the China-Myanmar border where the authors proposed the possible involvement of secondary and/or additional mechanisms in ART tolerance [35].

A reduction in sensitivity of *P. falciparum* isolates to ARTs was associated with mutations in the gene encoding *Pfcoronin* by Demas et al. [16]. The *Pfcoronin* SNPs (G50E, R100K, and E107V) that were implicated by Demas et al. were not detected in the present study [16]. However, this study detected a missense mutation (V424I) which is non-resident within the WD40 domain in D10 and D30 isolates with high ring survival rates. This mutation is new in Ghanaian isolates, though previously reported in a Peruvian population [37]. Its role in the observed high ring survival rates remains to be defined. The extent of ARTs and partner drug usage, the background mutations inherent in the parasite, and the selection pressures within local malaria parasite populations in Africa may vary from what pertains in SEA [38]. Therefore, correlates of ART tolerance in Ghanaian malaria parasite population and Africa at large, may vary from that of SEA.

Mutations in codons 86, 184, 1034, 1042, and 1246 of *Pfmdr1* gene have been involved in resistance to various anti-malarial drugs and the emergence of parasites having multidrug resistance [39]. The D650N and N652D mutations in *Pfmdr1* borne by one isolate with high ring survival in this study are novel in Ghanaian isolates but have been described by Ménard et al. in F32-ART5 (ART resistant) and F32-TEM (ART sensitive) Tanzanian isolates [40], while the Y184F and N86Y *Pfmdr1* mutations carried by the same isolate were reported to be associated with reduced sensitivity to piperaquine (PPQ) and AQ, which are ACT partner drugs [41, 42]. This same isolate harboured the *Pfmdr2* mutation (F423Y), which was earlier associated with in vitro pyrimethamine resistance [43]. Also borne by this isolate, were S208N and I492V *Pfmdr2* mutations whose roles in ART resistance are not known but have also been previously reported in African isolates [43, 44]. Furthermore, this isolate harboured N245S mutation and (ATT/ASN) deletion in the *Pfmdr2* gene, which have not yet been reported in Ghanaian malaria parasite population. Their potential effect in (or “contribution to”) drug tolerance remains to be elucidated.

Also, two of the isolates with RSA values > 10% (D10 & D30), each harboured D1951N mutation in the *Pfmrp2* gene and this is novel in Ghanaian isolates. The *Pfmrp2* gene on chromosome 12 encodes an ABC transporter which is mostly expressed at the ring stage of the malaria parasite [45, 46] and may have an association or potential for drug resistance. Nonetheless, the contribution of this novel mutation to the high RSA values observed, remains yet unknown.

An insertion (T) in the *P. falciparum* signal peptide peptidase (*PfSPP*) gene was detected in D10 (an isolate with high ring survival in this study). The *PfSPP* gene is essential for the survival and growth of the parasite in the erythrocytes of the human host [47, 48]. However, the role of this insertion in ring survival is currently unknown.

Additionally, mutations in *Pfdhps* (S436A, A581G, A613S, I431M) and *Pfdhfr* (N51I, C59R, S108N, D212I) genes, though not related to ART resistance, were explored in the present study. These mutations are still worth reporting due to their potential effect on sulfadoxine-pyrimethamine (SP) use of intermittent preventive treatment for malaria in pregnancy in the Ghanaian population. This is the first time that D212I and I431M substitutions in *Pfdhfr* and *Pfdhps*, respectively, are being reported in Ghanaian clinical isolates.

The IC_50s_ of D10 and D30 (isolates with > 10% ring survival) together with four isolates that had no ring survival after exposure to 700 nM DHA were measured to evaluate any potential association of ring survival with the IC_50s_ of ART, AS, AM, DHA, LUM, and AQ. In comparison to literature threshold values for drug resistance [28, 29, 49], the geometric mean IC_50s_ of all the drugs assayed could not establish drug resistance although the IC_50_ values of these drugs were marginally elevated than that described by Quashie et al. for 2012 from three sentinel sites (Cape Coast, Navrongo, and Hohoe) that suggests risen trends in AS IC_50s_ in Ghana [26]. Although IC_50s_ were measured for only few isolates, the observations made may be an indicator of a slow onset of drug-tolerance in the studied *P. falciparum* population over time.

There was no significant correlation between percent ring-stage-survival ex vivo and the reported IC_50s_ of DHA for the six isolates of interest. Results from the present study compare with the reports by Ikeda et al. [34] and Witkowski et al. [17] proposing the absence of significant association between increased ex vivo ring survival rates and the IC_50s_ to DHA in the conventional ex vivo drug sensitivity assay.

## Limitations

The study participants were recruited within a geographically limited area (Greater Accra Region of Ghana). Therefore, the generalizability of these findings may be limited. However, the outcome of this study may serve as grounds for continuous monitoring of ART tolerance in Ghanaian clinical isolates. Extension of follow up till day 28 could have provided additional information on parasite clearance and enabled in vivo efficacy assessment information to be done. Limited samples were used for the whole genome sequencing and IC_50_ assays (genotyping of three of the RSA negative isolates (P52, S22, S19) was not successful). The findings reported in the present study thus, require further studies with a much larger sample size.

## Conclusion

The very low proportion of individuals with day 3 post-treatment parasitaemia is consistent with rapid ART clearance in the studied patient population. However, the survival of some rings in the 72-h ex vivo ring stage survival test may be a sign of early development of ART tolerance in a portion of the parasite population under study. The IC_50_ values of the selected isolates were not indicative of drug tolerance/resistance, and no notable correlation was established between percent ring survival and IC_50_ values of RSA positive (> 10% survival rates) isolates and those with no ring survival. The genetic backgrounds noted in other drug resistance genes in isolates with > 10% ring survival calls for further inquiry to establish their combined effect on malaria parasite ring stage survival.

## Supplementary Information

**Additional file 1: ST1.** Primer details for Selective Whole Genome Sequencing (sWGS). **ST2.** The reaction mix for PCR products amplification for sWGS. **ST3.** Program: “Stepdown” protocol. **SF1.** Gel image of selected PCR products for sequencing, L = 1 kb ladder. **SF2.** The 2-fold serial dilution of test drug concentrations

## Data Availability

All data generated or analysed during this study are included in this published article and in the supplementary information.

## Abbreviations

ACT: Artemisinin-based combination therapy
AL: Artemether-Lumefantrine
AM: Artemether
AQ: Amodiaquine
ART: Artemisinin
CMP: Centre for Medical Parasitology
DHA: Dihydroartemisinin
DHC: Danfa Health Centre
Pfdhfr: Dihydrofolate reductase
GMIS: Ghana Malaria Indicator Survey
IC_50_: 50% Inhibition Concentration
INDELs: Insertions and Deletions
IVART: In vitro Analysis and reporting tool
PfK13: *P. falciparum* Kelch 13-propeller domain
LUM: Lumefantrine
MNPs: Multiple nucleotide polymorphisms
MSAT: Mass screening and treatment
NMIMR: Noguchi Memorial Institute for Medical Research
PCR: Polymerase Chain Reaction
Pfmdr1: *P. falciparum* Multidrug resistance protein 1
Pfcrt: *P. falciparum* Chloroquine transporter
Pfmrp2: *P. falciparum* Multidrug resistance-associated protein 2
Pfmdr2: *P. falciparum* Multidrug resistance protein 2
PfSPP: *P. falciparum* Signal peptide peptidase
PMLCH: Princess Marie Louise Children’s Hospital
Pfdhps: *P. falciparum* Dihydropteroate synthetase
RBCs: Red Blood Cells
RDTs: Rapid diagnostic tests
RSA: Ring-stage survival assay
SEA: Southeast Asia
SNPs: Single Nucleotide Polymorphisms
SODH: Shai Osudoku District Hospital
sWGS: Selective whole genome sequencing
WGS: Whole genome-sequencing
WWARN: Worldwide Antimalarial Resistance Network
WHO: World Health Organization
