# Supplementary material for: Assessment of artemisinin tolerance in Plasmodium falciparum clinical isolates in children with uncomplicated malaria in Ghana
Source: Malar J. 2023 Feb 19;22:58. doi: 10.1186/s12936-023-04482-w (PMC9938975; doi:10.1186/s12936-023-04482-w)
Supplement: Supplementary file 1 — Additional file 1: ST1. Primer details for Selective Whole Genome Sequencing (sWGS). ST2. The reaction mix for PCR products amplification for sWGS. ST3. Program: “Stepdown” protocol. SF1. Gel image of selected PCR products for sequencing, L = 1 kb ladder. SF2. The 2-fold serial dilution of test drug concentrations [file 12936_2023_4482_MOESM1_ESM.docx]

**SUPPLEMENTARY INFORMATION**

**How the 1L Culture medium was Prepared**

**RPMI powder (10.4g) was added to distilled water (850 ml) in a 1L sterile Winchester bottle having a magnetic stirrer. HEPES powder (7.15g) was added, followed by the addition of 2 g dextrose. Two milliliters of (25 mg/ml in NaOH) hypoxanthine was included in the mixture. Thirty-two (32 ml) of sodium bicarbonate (NaHCO3) and 2 ml of 10 mg/ml gentamicin was then added to the medium. The solution was completed by the addition of 25 ml of 0.5% Albumax as a replacement for inactivated human serum.** Thorough mixing of the medium was done using the magnetic stirrer after which sterile filters of 0.22 µm pore size were used to filter the medium. To avoid bacterial contamination, the filtration of the medium was done in a biosafety cabinet. The hypoxanthine solution was made by dissolving 0.25 g hypoxanthine powder in 10 ml of NaOH. The Albumax solution was prepared by dissolving Albumax powder (100g) in incomplete RPMI medium (500 ml) while the NaHCO_3_ solution was made by dissolving NaHCO_3_ (37.5g) in distilled water (500 ml).

**How the DHA solution was prepared**

One milligram per milliliter (1 mg/ml) stock solution was made by the resuspension of a WWARN-acquired ~1 mg pre-weighed aliquot of DHA. Thorough dissolution was achieved by vortexing the mixture. To obtain a 200 µg/ml solution of DHA, dissolution of the stock DHA solution (a 5-fold) was made and vortexed. Various aliquots of 50 µl were made and stored at -20^o^C in sterile 1.5 ml microcentrifuge tubes. These aliquots were not used beyond 6 months.

**How the uninfected RBCs were prepared.**

The uninfected RBCs were obtained from blood group O+ colleagues in the epidemiology laboratory of Noguchi Memorial Institute for Medical Research (NMIMR) after negative RDT and microscopy test results. We aseptically collected venous blood samples from haemoglobinopathies-free and G6PD-functional volunteer donors using Vacutainer needles, into 8 ml ACD tubes. Transferred into 15 ml Eppendorf tubes in a biosafety cabinet, the samples got centrifuged at 1000 g for 5 minutes. Cold RPMI was added to the cells after the removal of the plasma and buffy coat and centrifuged for 5 minutes at 1000 g (washing done 3 times). Fifty percent (50%) haematocrit was achieved by adding an equal volume of RPMI to the washed RBCs. The resultant uninfected RBCs was stored for up to 4 days at 4^o^C.

**How the Giemsa stain was prepared and used to stain blood smears**

To nine milliliters (9 ml) of distilled water was added a 1 ml volume of Giemsa stock solution (Merck). Blood smears were stained for 15 minutes with the resultant working Giemsa solution (10%). Microscopic examination of blood smears was done under oil-immersion after the stain was rinsed off the smears and the smears were air-dried.

**How blood was collected from study participants and prepared**

With the use of a Vacutainer safety-Lok blood collection set and a holder, venous blood samples were aseptically drawn into an ACD vacutainer. Sample clotting was prevented by gently inverting the blood tubes five times to mix the blood with the anticoagulant. The samples were **stored at 4^o^C and transported in ice chest to the NMIMR lab via a project motorbike for the start of the RSA within 24 hours.** Thin blood smears were made (from a drop of blood) and parasitaemia was evaluated after the smears were Giemsa-stained. Plasma and buffy coat were removed from the blood samples contained in 15 ml Eppendorf tubes. This was done in a biosafety cabinet after the tubes were centrifuged for 5 minutes at 800 g. The tubes were again centrifuged at 800 g for 5 minutes, twice after the addition of pre-warmed RPMI at room temperature (25°C). The parasite-infected RBCs were thus ready for the *ex vivo* RSA.

**How the drug solution was prepared**

The DMSO control solution was prepared by adding 2 ml of culture medium to 20 µl of stock DMSO (to give a 0.1% DMSO for the drug-free well). Twenty microlitres (20 µl) of DHA stock solution was added to 2 ml of culture medium to constitute the DHA test solution. The drug solutions were thoroughly mixed by vortexing. Once thawed, the DHA aliquots were used only once and not reused.

**How parasite survival rates were determined by microscopy**

A thin slide was prepared at 0 hours and labelled “INI” for initial pretreatment parasitaemia. Another thin slide was prepared from the DMSO control well at 72 hours labelled “NE” for the non-DHA exposed parasitemia. Additionally, a third thin slide was made at 72 hours for the DHA-exposed parasitaemia. The 3 thin slides were stained and examined under oil-immersion at 100× magnification. Per standard procedure, in a total of 10,000 RBCs, only parasitized RBCs having viable parasites were counted. Determination of the proportion of viable parasites in DHA and “NE” (percent survival) was done as described by Witkowski et al. (1) Counting was done in portions of the slide that contain about 200-400 RBCs per field and not at the edge of the smears.

**Details of the Primers used for sWGS PCR**

**ST1:** Primer details for Selective Whole Genome Sequencing (sWGS)

| **Primer name** | **Primer sequence** | **Primer quantity ordered** | **Primer formulation** |
| --- | --- | --- | --- |
| **Pf10** | TATTATATA*T | 250 nmole | STD |
| **Pf9** | AATAATAATA*A | 250 nmole | STD |
| **Pf8** | AAAAAAAAAAA*A | 250 nmole | STD |
| **Pf7** | TAATAATAAT*A | 250 nmole | STD |
| **Pf6** | ATTATTATTA*T | 250 nmole | STD |
| **Pf5** | TATATATATT*T | 250 nmole | STD |
| **Pf4** | TAATATATA*T | 250 nmole | STD |
| **Pf3** | TATATATATA*A | 250 nmole | STD |
| **Pf2** | TATATATATAT*T | 250 nmole | STD |
| **Pf1** | ATATATATAT*A | 250 nmole | STD |
|  | | | |

* Phosphorothioate bond. STD: standard desalting purification

| **Reagents** |  | **Vol. per sample** | **Vol. in mix** | **Final conc. per sample** |
| --- | --- | --- | --- | --- |
| **_H2O_** |  | 13.5 µl | **^2^** |  |
| 5 µM of each primer |  | 25 µl | **^Primers^** | 2.5 µM |
| 30 U Phi29 | 10 U/µl | 3 µl |  |  |
| 1x Phi29 buffer | 10× | 5 µl |  |  |
| 1mM dNTPs |  | 2 µl |  | 1mM |
| 1x BSA (100X) |  | 0.5 µl |  | 1x BSA |

**ST2:** The reaction mix for PCR products amplification for sWGS

To a 49 µl Master Mix was added 1 µl 5 ng sample DNA for a total reaction capacity of 50 µl. The sequencing reaction for the isolates of interest was carried out in 0.2 ml PCR-tubes. dNTP, deoxynucleoside triphosphate. BSA, bovine serum albumin

**ST3.** Program: “Stepdown” protocol

35°C for 5 min

33°C for 15 min

32°C for 20 min

31°C for 30 min

30°C for 16 h

65°C for 15 min

Results of the targeted PCR extension are as shown below:


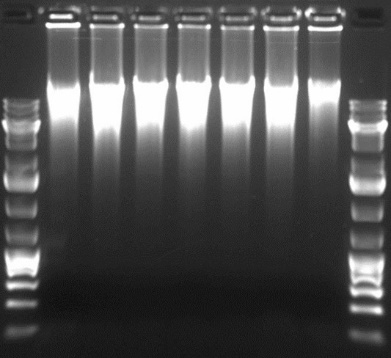
 L P11 36 32 D10 30 P55 45 L

**SF1.** Gel image of selected PCR products for sequencing, L = 1 kb ladder

**How the stock concentrations of the drugs were prepared**

Artemisinin and its derivatives were prepared in DMSO while methanol was used to prepare LUM and AQ stock solutions. Five milligrams (5 mg) of pure DHA powder was added to 1 ml of DMSO to prepare a DHA stock solution of 5,000,000 ng/ml upon dissolution. For an AM stock concentration of 1,000,000 ng/ml, 1 mg of purified AM powder was added to 1 ml of DMSO and vortexed to dissolve. To prepare an ART stock solution of 10,000,000 ng/ml, 10 mg of purified ART powder was added to 1 ml of DMSO and vortexed to dissolve. To make a stock solution of AS 15,000,000 ng/ml, 15 mg of purified AS powder was added to 1 ml of DMSO and stirred to dissolve. To prepare an AQ stock solution of 10,000,000 ng/ml, 10 mg of purified AQ powder was added to 1 ml of methanol and vortexed to dissolve. To make a LUM stock solution of 1,000,000 ng/ml, 1 mg of LUM powder was added to 1 ml of methanol and vortexed to dissolve.

**How start / working concentrations were prepared**

The formula C_1_V_1_ = C_2_V_2_ was used to prepare the working solutions of the drugs from their respective stock solutions. For instance, to prepare 10 ml of 400 ng/ml of DHA drug solution from a stock solution of 5,000,000 ng/ml, the volume of stock required, V_1_ = 400 × 10 / 5,000,000 = 0.0008ml or 0.8 µl. Therefore, 0.8 µl of the drug stock was added to 9 ml, 999.2 µl media. Using similar approach, working drug volumes 5 ml, 10 ml, 30 ml, 20 ml, and 5 ml of 2,000 ng/ml, 1000 ng/ml, 400 ng/ml, 400 ng/ml, and 400 ng/ml were made from 1,000,000 ng/ml, 10,000,000 ng/ml, 15,000,000 ng/ml, 10,000,000 ng/ml, and 1,000,000 ng/ml stocks for LUM, AQ, AS, ART, and AM, respectively.

**How the 2-fold serial dilution of the drugs was prepared**

The working drug solutions (400 ng/ml for ART and its derivatives; 2,000 ng/ml for LUM; and 1,000 ng/ml for AQ) were 2-fold serially diluted to lower concentrations for the drug sensitivity assays (ARTs: 0.78-200 ng/ml; LUM: 3.91-1,000 ng/ml; AQ: 1.95-500 ng/ml). In summary, 100 µl each of the test drugs were taken from well 1 into well 2 which contains 100 µl of media. This was mixed well and 100 µl of the resultant drug solution was taken into well 3 and so on. One hundred µl of the resultant drug solution were discarded after the contents in well 9 were mixed.

**
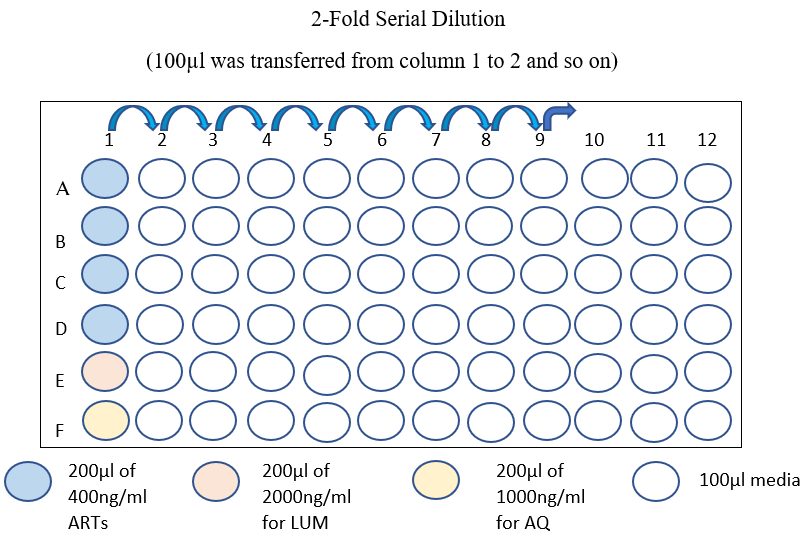
SF2.** The 2-fold serial dilution of test drug concentrations

The pre-dosed drug plates were prepared in triplicates for each drug of interest as follows:

Media: incomplete media (to prevent evaporation), CPM: complete media (control well)

Sample ID: X

| Plate 1 |  | 1 | 2 | 3 | 4 | 5 | 6 | 7 | 8 | 9 | 10 | 11 | 12 |
| --- | --- | --- | --- | --- | --- | --- | --- | --- | --- | --- | --- | --- | --- |
|  | A | media | media | media | media | media | media | media | media | media | media | media | media |
| ART | B | media | 200 | 100 | 50.0 | 25.0 | 12.5 | 6.25 | 3.13 | 1.56 | 0.78 | CPM | media |
| ART | C | media | 200 | 100 | 50.0 | 25.0 | 12.5 | 6.25 | 3.13 | 1.56 | 0.78 | CPM | media |
| ART | D | media | 200 | 100 | 50.0 | 25.0 | 12.5 | 6.25 | 3.13 | 1.56 | 0.78 | CPM | media |
| AS | E | media | 200 | 100 | 50.0 | 25.0 | 12.5 | 6.25 | 3.13 | 1.56 | 0.78 | CPM | media |
| AS | F | media | 200 | 100 | 50.0 | 25.0 | 12.5 | 6.25 | 3.13 | 1.56 | 0.78 | CPM | media |
| AS | G | media | 200 | 100 | 50.0 | 25.0 | 12.5 | 6.25 | 3.13 | 1.56 | 0.78 | CPM | media |
|  | H | media | media | media | media | media | media | media | media | media | media | media | media |

Sample ID: X

| Plate 2 |  | 1 | 2 | 3 | 4 | 5 | 6 | 7 | 8 | 9 | 10 | 11 | 12 |
| --- | --- | --- | --- | --- | --- | --- | --- | --- | --- | --- | --- | --- | --- |
|  | A | media | media | media | media | media | media | media | media | media | media | media | media |
| AM | B | media | 200 | 100 | 50.0 | 25.0 | 12.5 | 6.25 | 3.13 | 1.56 | 0.78 | CPM | media |
| AM | C | media | 200 | 100 | 50.0 | 25.0 | 12.5 | 6.25 | 3.13 | 1.56 | 0.78 | CPM | media |
| AM | D | media | 200 | 100 | 50.0 | 25.0 | 12.5 | 6.25 | 3.13 | 1.56 | 0.78 | CPM | media |
| DHA | E | media | 200 | 100 | 50.0 | 25.0 | 12.5 | 6.25 | 3.13 | 1.56 | 0.78 | CPM | media |
| DHA | F | media | 200 | 100 | 50.0 | 25.0 | 12.5 | 6.25 | 3.13 | 1.56 | 0.78 | CPM | media |
| DHA | G | media | 200 | 100 | 50.0 | 25.0 | 12.5 | 6.25 | 3.13 | 1.56 | 0.78 | CPM | media |
|  | H | media | media | media | media | media | media | media | media | media | media | media | media |

Sample ID: X

| Plate 3 |  | 1 | 2 | 3 | 4 | 5 | 6 | 7 | 8 | 9 | 10 | 11 | 12 |
| --- | --- | --- | --- | --- | --- | --- | --- | --- | --- | --- | --- | --- | --- |
|  | A | media | media | media | media | media | media | media | media | media | media | media | media |
| LUM | B | media | 1000 | 500 | 250 | 125 | 62.5 | 31.25 | 15.63 | 7.81 | 3.91 | CPM | media |
| LUM | C | media | 1000 | 500 | 250 | 125 | 62.5 | 31.25 | 15.63 | 7.81 | 3.91 | CPM | media |
| LUM | D | media | 1000 | 500 | 250 | 125 | 62.5 | 31.25 | 15.63 | 7.81 | 3.91 | CPM | media |
| AQ | E | media | 500 | 250 | 125 | 62.5 | 31.25 | 15.63 | 7.81 | 3.91 | 1.95 | CPM | media |
| AQ | F | media | 500 | 250 | 125 | 62.5 | 31.25 | 15.63 | 7.81 | 3.91 | 1.95 | CPM | media |
| AQ | G | media | 500 | 250 | 125 | 62.5 | 31.25 | 15.63 | 7.81 | 3.91 | 1.95 | CPM | media |
|  | H | media | media | media | media | media | media | media | media | media | media | media | media |

**How the parasite mix was prepared**

One percent (1%) parasitaemia in 2% haematocrit was prepared for the samples as shown below:

Foremost, C_1_V_1_ = C_2_V_2_ was used to calculate the 2% haematocrit, where C_1_ = 50% (denotes uninfected O+ RBCs), and the overall volume of RBCs required for the preparation was V_1._ The desired haematocrit to be prepared was C_2_ and the total volume of the parasite mixture needed to plate all 6-drug panel was V_2_. Therefore, for the preparation of 13 ml of 2% haematocrit, V_1_ = C_2_V_2_ / C_1_, i.e., 2 × 13 / 50 = 0.52 ml or 520 µl (*this was multiplied by 2 to arrive at 1,040 µl). So, the total RBCs (including uninfected and parasitized RBCs) needed in the final parasite mix was 1,040 µl.

Additionally, C_1_V_1_ = C_2_V_2_ was used to compute the 1% parasitaemia. For instance, to reduce a sample with an initial parasitaemia of 6.3% to 1%, the volume of parasitized RBCs needed in the mix was V_1_ = 1 x 1,040 / 6.3 = 0.165 ml or 165 µl (where C_1_ = 6.3%, C_2_ = 1%, and V_2_ = 1,040 µl). This makes the volume of uninfected RBCs needed in the final mix 1,040-165 µl = 875 µl, and the complete media volume required in the final mix 13,000-1040 µl = 11,960 µl or 11 ml, 960 µl.

*The rationale for this was to maintain the percent parasitaemia and haematocrit at 1% and 2%, respectively, after the addition of 100 µl of the parasite mix to 100 µl of drug solution.

**How the SYBR Green 1-lysis buffer solution was prepared.**

The MSF lysis buffer solution was prepared by the dissolution of 2.423 g of Tris base in 1 L double distilled water (20 mM_final conc_.) using a magnetic stirrer. The pH of the buffer was then adjusted to 7.5 using concentrated HCl. To this solution was added 10 ml 0.5 M EDTA (5 mM_final conc._), 80 mg saponin (0.008% w/v final), and 0.8 ml Triton X-100 (0.08% w/v final) and homogenously mixed, avoiding bubbles. This solution was stored at room temperature after filtration. To the MSF lysis buffer was added thawed -80^o^C frozen aliquots of 10 000× SYBR Green 1 stock solution and evenly mixed while avoiding bubbles. Two microliters (2 µl) of SYBR Green I stock solution mixed with 10 ml of MSF lysis buffer (i.e., 0.2 µl SYBR Green I / ml of lysis buffer) is just about enough for a 96 well plate.

References

1. Witkowski, B, Menard D, Amaratunga C, Fairhurst RM. Ring‐stage Survival Assays (RSA) to evaluate the in‐vitro and ex‐vivo susceptibility of Plasmodium falciparum to artemisinins. Institute Pasteur du Cambodge – National Institutes of Health Procedure RSAv1. 2013.
